# Supplementary material for: Core Microbiome and Microbial Community Structure in Coralloid Roots of Cycas in Ex Situ Collection of Kunming Botanical Garden in China
Source: Microorganisms. 2023 Aug 24;11(9):2144. doi: 10.3390/microorganisms11092144 (PMC10537389; doi:10.3390/microorganisms11092144)
Supplement: Supplementary file 1 [file microorganisms-11-02144-s001.zip › Supplementary Figure.pdf]

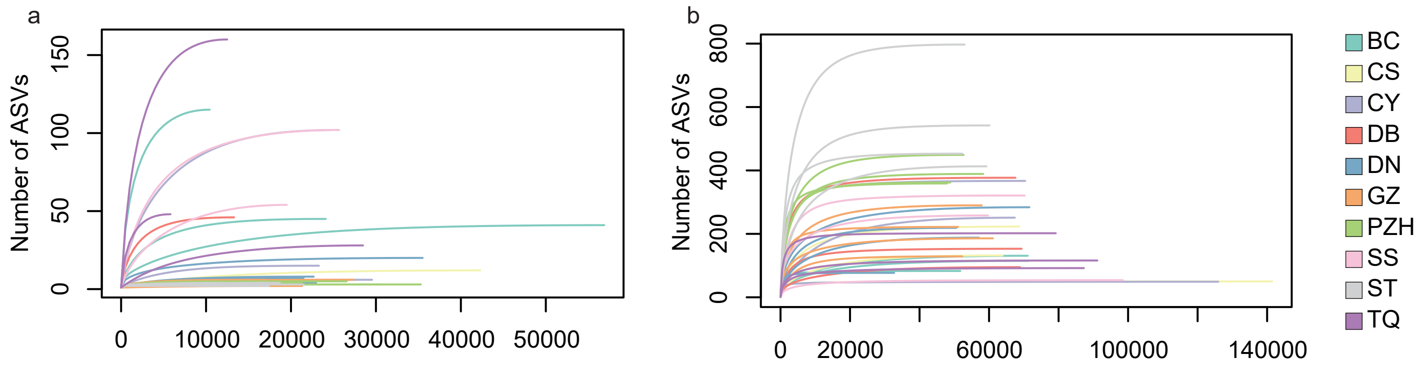

Figure S1. Rarefaction curves of 34 samples of *Cycas* revealed by 16sRNA(a) and ITS sequence (b).

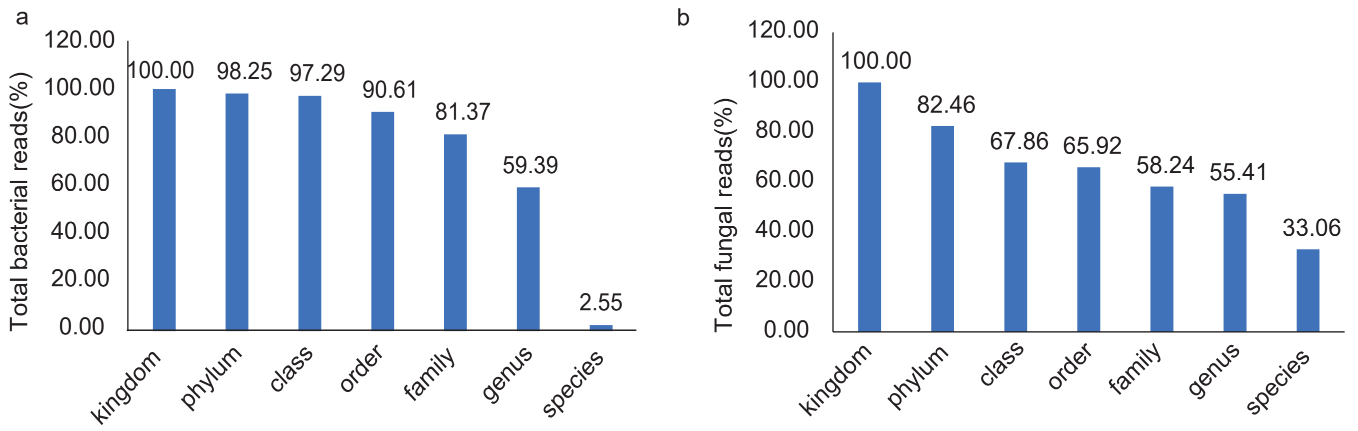

Figure S2. The proportion of endophytic bacteria (a) and fungi (b) of *Cycas* samples assigned to taxonomical ranks.

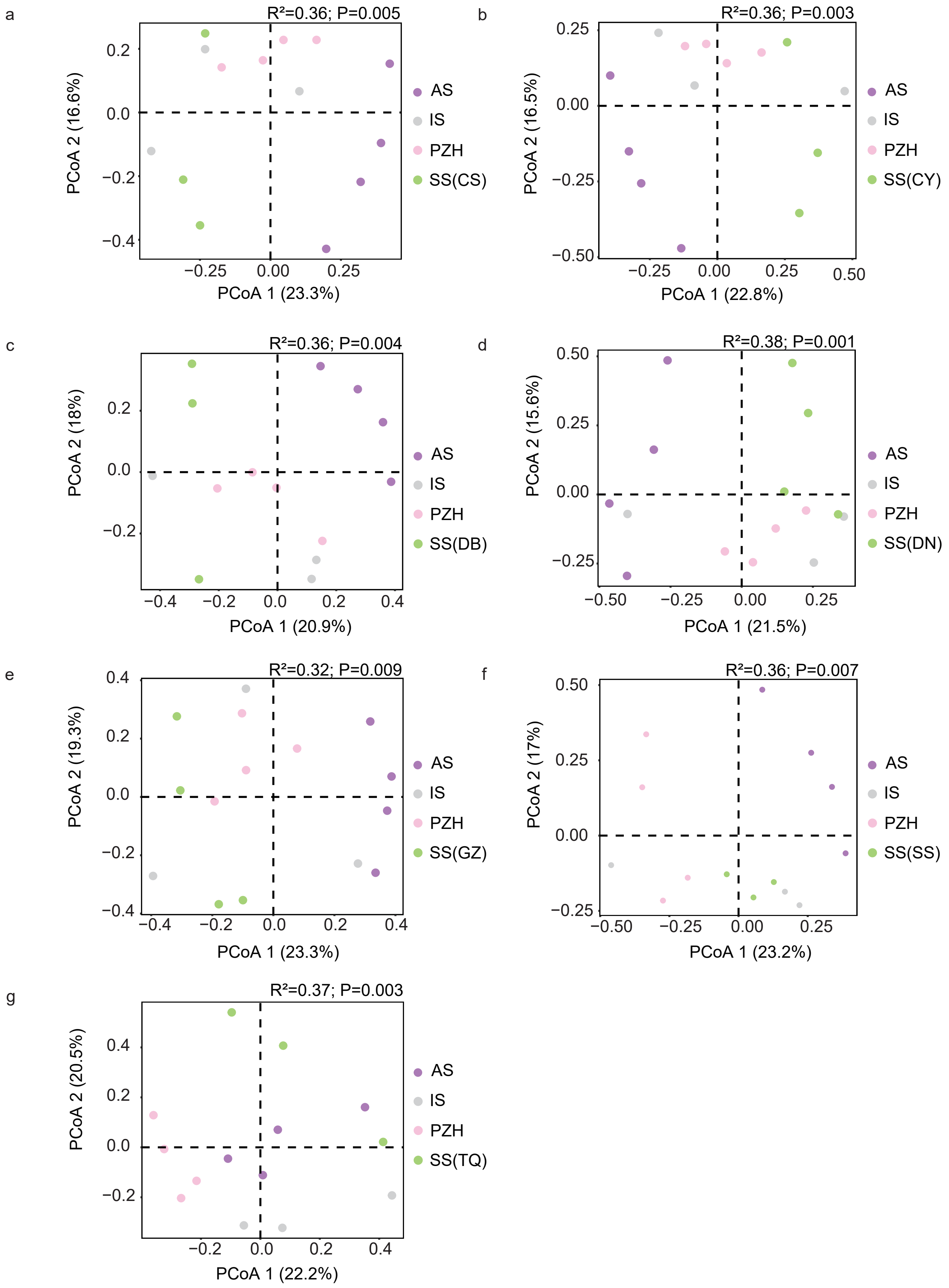

Figure S3. PCoA analyses using samples from every species of the sect. *Stangerioides* with three other sections, respectively.
